# Color Centers Enabled by Direct Femto-Second Laser Writing in Wide Bandgap Semiconductors

**DOI:** 10.3390/nano11010072

**Published:** 2020-12-31

**Authors:** Stefania Castelletto, Jovan Maksimovic, Tomas Katkus, Takeshi Ohshima, Brett C. Johnson, Saulius Juodkazis

**Affiliations:** 1 School of Engineering, RMIT University, Melbourne, VIC 3000, Australia; 2 Optical Sciences Center and ARC Training Centre in Surface Engineering for Advanced Materials (SEAM), Swinburne University of Technology, John Street, Hawthorn, VIC 3122, Australia; jmaksimovic@swin.edu.au (J.M.); tkatkus@swin.edu.au (T.K.); sjuodkazis@swin.edu.au (S.J.); 3 National Institutes for Quantum and Radiological Science and Technology, 1233 Watanuki, Takasaki 370-1292, Japan; ohshima.takeshi@qst.go.jp; 4 Centre for Quantum Computation and Communication Technology, School of Physics, The University of Melbourne, Melbourne, VIC 3001, Australia; johnsonb@unimelb.edu.au; 5 Tokyo Tech World Research Hub Initiative (WRHI), School of Materials and Chemical Technology, Tokyo Institute of Technology, 2-12-1, Ookayama, Meguro-ku, Tokyo 152-8550, Japan

**Keywords:** femtosecond laser writing, color centers, vacancies, silicon carbide, gallium nitride, spectroscopy

## Abstract

Color centers in silicon carbide are relevant for applications in quantum technologies as they can produce single photon sources or can be used as spin qubits and in quantum sensing applications. Here, we have applied femtosecond laser writing in silicon carbide and gallium nitride to generate vacancy-related color centers, giving rise to photoluminescence from the visible to the infrared. Using a 515 nm wavelength 230 fs pulsed laser, we produce large arrays of silicon vacancy defects in silicon carbide with a high localization within the confocal diffraction limit of 500 nm and with minimal material damage. The number of color centers formed exhibited power-law scaling with the laser fabrication energy indicating that the color centers are created by photoinduced ionization. This work highlights the simplicity and flexibility of laser fabrication of color center arrays in relevant materials for quantum applications.

## 1. Introduction

Femtosecond (fs) lasers are well-developed tools for ablation and micro-/nanofabrication and have been used for 3D direct patterning and micromachining of transparent optical materials [1]. It is possible to form nanoscale bumps on glass surface by controlled re-melting with nJ-energy pulses [2], to write 3D pattern of submicrometer-sized modifications inside silica for 3D optical memory, which can withstand temperatures of ~1000 °C [3], and to induce crystalline to amorphous phase transitions within subwavelength volumes as demonstrated with sapphire [4,5]. Even higher nanoscale precision was demonstrated in nanoscale ablation of ripples [6], which ultimately can be used for direct writing of nanogrooves of tens-of-nm for lithography applications [7] and could be used for patterning complex 3D surfaces. The localization of nanoscale ablation and 3D modification of materials is controlled by the polarization of laser pulse via E-field enhancement due to nanoscale-localized permittivity change between excited/ablated and pristine material, which defines strongly nonlinear light–matter interaction [8]. Recently, we demonstrated that surface and volume patterning at high industrial writing speeds can be achieved without stitching errors caused by connection of individual writing fields [9]. Currently, fs-laser inscription of nanoscale structures is a ready technique for large area applications.

In particular, fs-laser writing has been proposed as a method to create vacancies giving rise to photoluminescence (PL) in hard transparent materials and wide bandgap semiconductors such as diamond [10,11,12,13], silicon carbide (SiC) [14,15], cubic boron nitride (cBN) [16], 2D hexagonal BN [17], and thin films of gallium nitride (GaN) on sapphire and nanowires [18]. The PL of these defects can be studied at room and low temperature using Raman spectroscopy and customized fluorescence confocal microscopy methods equipped with single photon detectors. These systems are sensitive to the very localized fabrication areas and can yield information at the single-color center level formed by fs-laser writing methods.

Additionally, these materials have recently emerged as platforms for hosting color centers associated to deep-level defects in the bandgap, giving rise to single photon sources [19,20,21,22,23] and providing optical read-out of their electron spin for applications in quantum spintronics [24,25,26]. Facile fabrication methods of these hard materials are also relevant for advances in quantum photonics and in particular for on-chip integration if the required localization and yield of formation could be achieved [27]. As such the identification of color centers fabricated by femtosecond laser is significant to establish this method as a facile, on demand, and localized procedure with 3D potentials, where damage to the crystal lattice is minimized.

The materials mostly studied involving color centers formation using femtosecond laser fabrication are diamond, followed by, boron nitride, and silicon carbide.

In diamond, the silicon vacancy [11] and the nitrogen vacancy (NV) color centers [10,11,12,13] were identified after the laser writing fabrication. Using the fs-laser to both create and diffuse defects via local annealing inside a nitrogen-doped diamond, single NV could be created and detected in real time yielding a close to deterministic formation control [28].

In SiC, direct laser writing permits the creation of the silicon vacancy, *V*_Si,_ with emission in the spectral region 850–950 nm [14,15], while oxygen-related vacancies with a white light emission [29] were demonstrated using picosecond lasers. Waveguides in SiC have also been achieved using femtosecond lasers [30].

In GaN, emission from 620 (thin layer) to 680 nm wavelengths, depending on the material, was shown; no assignment to specific color centers was provided, whereas a general red luminescence (RL) between 1.5 ÷ 2 eV in GaN is reported in reference [31] and unclearly identified, possibly related to a complex of *V*_Ga_. This is consistent with defect-related PL in GaN-based materials [32]. A tentative assignment to an emission with peak at 689 nm in GaN was also attributed to a transition from conduction band to a deep acceptor of unknown origin [33].

PL observed after laser writing can be attributed to a mechanism of vacancies formation in transparent wide-bandgap materials. For a transparent wide bandgap material irradiated by an intense femtosecond laser, the density of photoexcited free electrons can reach critical plasma density and dielectric breakdown, which leads to defect generation including vacancies, e.g., as observed in sapphire [34].

In this work, we focus on the fabrication and PL characterization of color centers formed by fs-laser writing via confocal imaging and spectroscopy in high-purity semi-insulating (HPSI), intrinsic unintentionally doped and n-doped SiC, and unintentionally doped GaN films. We used a laser with pulse temporal width of 230 fs with central wavelengths of 1030 and 515 nm, which delivered a single pulse at each writing site. Various energies per pulse were investigated and high vacancy localization in some cases within the diffraction limit of the confocal microscope was achieved. We find that 515 nm is a better choice in terms of fabrication localization compared to 1030 nm. From low temperature PL characterization, we confirm the formation of silicon vacancies through the identification of the *V*1′ line in high purity semi-insulating SiC and we also show the formation of a 665 nm emission, which is not assigned. We did not observe the formation of silicon vacancies in highly n-doped SiC, while we have observed via confocal mapping, an emission at longer wavelength, which is also not currently assigned. In regards to GaN, we observed a laser energy-dependent emission, which could be due to a *V*_Ga_, however, a blue shift of the emission is observed by higher energy writing.

## 2. Light–Matter Interaction

In a transparent material, there is no linear absorption of the incident laser light. To achieve optical breakdown and material damage, nonlinear absorption mechanisms, which result in photoionization or the promotion of electrons from the valence band to the conduction band, occur. This can take place via multiphoton ionization (MPI) and the tunneling ionization (Zener breakdown).

In the tunneling ionization regime (for large laser field and low laser frequency), the electron tunnels through the atomic potential barrier and becomes free, while in the MPI regime, N photons are consumed to generate one free electron so that the total energy absorbed is equal to or greater than the bandgap ($E_{g}$) of the material in order to transfer electrons into the conduction band, $\hbar \omega N\geq E_{g}$ [35]. In the MPI regime, the photoionization rate is $P\left(I\right)=\sigma_{N}\;I^{N}$, where $\sigma_{N}$ is the multiphoton absorption coefficient for N photons and I is the laser intensity.

The tunneling rate scales more weakly with the laser intensity than the MPI rate. Typically for pulse durations greater than 10 fs, the electrons excited by nonlinear absorption undergo a phonon-mediated linear absorption. The extra kinetic energy acquired by electrons is consumed to break chemical bonds (bound electrons) and to excite electrons to the conduction band, thus producing an avalanche ionization. The avalanche ionization requires seed electrons in the conduction band of the material and depends only linearly on I [1].

The dominant mechanism in the nonlinear absorption regime for free-electron generation is typically delineated by the Keldysh parameter [14,36], which is given by $\gamma =\frac{\omega }{e}\sqrt{\frac{m\;c\;n\;\varepsilon_{o}E_{g}}{2\;I}}$, where ω is the laser angular frequency, e,m is the electron charge and the reduced mass, respectively, n is the medium refractive index, $\varepsilon_{o}$ is the permittivity of free space, and I is the laser irradiance at the focal plane (denoted by the beam waist $w_{o}=\frac{4\;\lambda f}{\pi D}\approx \frac{2\;\lambda }{\pi NA}$ of the objective with numerical aperture NA). When γ>1.5, photoionization is dominated by MPI, otherwise, it is due to tunneling ionization. In the intermediate regime, the photoionization is a mix between tunneling and MPI. For laser intensities $I\leq I^{th}=\frac{m\;c\;n\;\varepsilon_{o}E_{g}\omega^{2}}{{\left(1.5*e\right)}^{2}}$, the MPI regime is prevalent where the $I^{th}$ is the threshold intensity to create vacancies. At $I<I^{th}$, fewer vacancies will be produced with a decreasing intensity/irradiance per pulse. It has been found that there is in practice a lower dependence on the bandgap energy of the $I^{th}$ as this can also depend on avalanche photoionization, and thus femtosecond laser micromachining can be used in a broad range of materials [1].

The mechanism resulting in the formation of known color centers in diamond and SiC has been attributed recently to photoionization process in the MPI regime [10,14], where only few to single color centers were created, as the creation of vacancies was achieved by high numerical aperture objectives to improve localization inside the material at 20–50 μm depth from the surface. A spatial light modulator (SLM) was used to correct for the aberration due to the high refractive index of the host which creates strong spherical aberration [37]. The number of photons absorbed in diamond and SiC were estimated to be 9 [38] and 16 photons [14], respectively.

In ref. [15], the creation of color centers in SiC at depths up to 10 μm from the surface may be attributed to the intermediate regime between MPI and tunneling ionization due to longer fabrication wavelength, to the much higher laser energy used, and only ensemble of *V*_Si_ were generated.

Previous laser fabrication experiments of NV in diamond were based on the use of high energy laser pulses, which produced a strong damage in the diamond, inducing graphitization and ablation in the irradiated zone [39], while NV centers were created in the vicinity of laser graphitized regions on the surface [40] and in the bulk [41]. In ref. [40], the formation of NV centers in diamond using intense ultrashort laser pulses has been attributed to nonlinear photoionization process such as MPI and tunneling ionization of oxygen and nitrogen molecules in air, generating free electrons strongly accelerated by the subsequent laser pulses. In this case, single NV centers were formed near the surface of the diamond. Another mechanism to form NV centers in diamond using fs laser was achieved in the regime of nanoablation <1 J/cm^2^ [42] with a logarithmic increase in NV center density with laser pulse number.

It is currently understood that the MPI regime is the most favorable to achieve the highest localization and controllably produce only one to a few emitters as required for quantum applications, while other regimes more likely produce ensembles of defects and single defects appear to be generated around the damaged area with no control of their location. However, the nanoablation has demonstrated a good localization close to the diamond surface and can be utilized to form defect arrays [42].

## 3. Materials and Methods

### 3.1. Laser Inscription

For the laser writing, a solid state Yb:KGW laser (Pharos, Light Conversion, Ltd., Vilnius, Lithuania) at central 1030 nm wavelength and its second harmonic at 515 nm, 230 fs duration, and repetition rate of 200 kHz was used. High precision mechanical stages (Aerotech, GmbH, Pittsburgh, PA, USA) were used for in-plane (*xy*) translation of the sample. A software–hardware integration solution of the entire fs-fabrication unit was made by Altechna Ltd., Vilnius, Lithuania.

For each writing site, a single laser pulse was delivered. The laser writing was applied to commercial on-axis, research-grade, high-purity semi-insulating (HPSI) 4H-SiC from CREE, unintentionally doped semi-insulating 4H-SiC, and an n-doped commercial 4H-SiC from SiCrysta. The GaN sample was an unintentionally n-doped thin wurtzite film grown on a sapphire substrate via metal-organic chemical vapor deposition (MOCVD). Details are provided in Table 1.

The writing objective lens has a 100× magnification and numerical aperture NA = 0.90 with a working distance of 1.3 mm. We assume a laser field intensity I(r, z) Gaussian profile, with waist $w_{o}$ (364 and 729 nm for SiC) and Rayleigh length $z_{R}=\frac{\pi w_{o}^{2}}{\lambda }\;$= 810 and 1619 nm, respectively. The focal volume is given by $V=\pi \;\ln2\;z_{R}w_{o}^{2}.$ The writing volumes based on a refractive index of 2.59 are approximately 0.23 and 1.9 μm^3^ for the visible and NIR writing wavelength, respectively. According to the nonlinearity of the absorption process, the vacancy generation will occur within a region smaller than the focal volume depending on the number of photons N absorbed in the MPI process to free electrons and modifying the volume as $V=\pi \;\frac{\ln2}{N}\;\sqrt{2^{1/N}-1\;}z_{R}w_{o}^{2}$. For SiC *V*_Si_ generation, it has been estimated that the process could occur by absorbing *N* = 16 [14], as such, the writing volume could be as small as 3 × 10^−3^ and 2.5 × 10^−2^ μm^3^. It is to be noted that this model is valid for electrons excitation to the conduction band based on nonlinear absorption of N photons, however, the mechanism of vacancies formation from the free electrons is not yet fully understood or modeled.

The laser writing pattern using 1030 nm was a 10 × 7 array of dots separated from each other by 5 μm with each line corresponding to tuned laser energy from 230 to 4 nJ per pulse focused on the GaN and SiC surfaces. The laser writing pattern using 515 nm on SiC was a large area array with dots written with energy per pulse in each line from 13 to 445 nJ at 2 and 4 μm depths, and the dots were written laterally with period of 8 μm.

**Figure 1:**
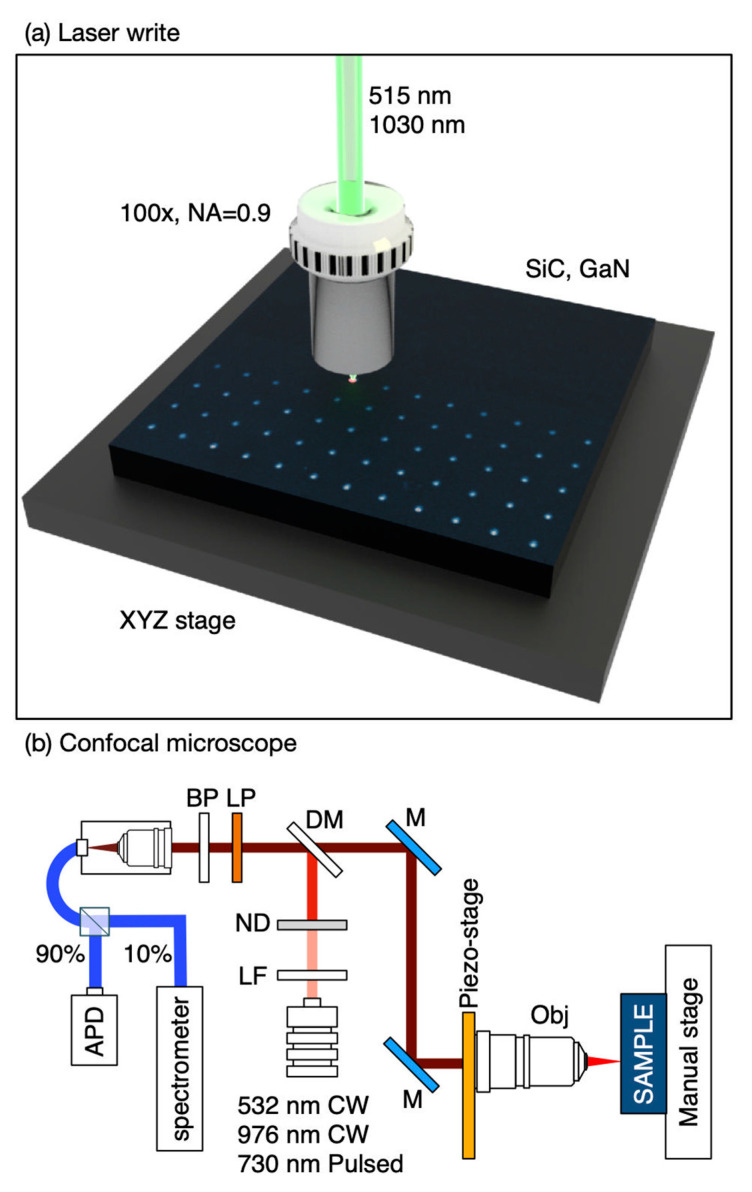
(**a**) Conceptual drawing of the laser writing system for color center creation. (**b**) Schematic diagram of the confocal fluorescence microscope.

Before laser writing, the SiC samples were cleaned with a Piranha solution to remove organic contaminants, and then annealed in an Argon atmosphere at 1000 °C for 1 h to reduce the as-grown *V*_Si_. For the characterization, we used Raman spectroscopy at low temperature and fluorescence microscopy. The experimental schematics for laser writing and fluorescence mapping are shown in Figure 1a,b, respectively.

### 3.2. Photoluminescence Mapping

To achieve PL maps, we have used two custom-made confocal systems, the first optimized to investigate *V*_Si_ emission equipped with 532 and 730 nm lasers and avalanche single-photon detector (APD, SPCM-AQRH-15, Excelitas Technologies, Waltham, MA, USA); the second with an excitation laser at 976 nm for the detection of emission in the near infrared using InGaAs avalanche single-photon detectors (ID-Quantique 230 Peltier cooled to −90 °C, quantum efficiency maximum of 25% at 1300 nm and set at 10% efficiency during measurement and deadtime of 2 μs).

With the 532 nm excitation laser, a dichroic mirror (DM) at 532 nm (LPD02-532RU-25), a notch filter (NF03-532E-25), and a short pass at 842 nm (FF01-842/SP-25) were used to study GaN laser written samples. The microscope objective was a Nikon (Tokyo Japan) 100× 0.9 NA with a 2 mm focal length.

In the same confocal a variable wavelength supercontinuum NKT Photonics (Birkerød, Denmark) Fianium WhiteLase pulsed laser was used for sample illumination at 730 nm to detect color centers in SiC nm (25 nm bandwidth) and 80 MHz. The laser power was finely controlled using a neutral density filter wheel (ND). An Olympus dry objective (100×, 0.85 NA) LCPLN-IR with 85% transmission at 900 nm was used. The objectives were mounted on a PI XYZ computer-controlled stage with an XYZ closed-loop positioner with a 200 μm travel distance in each direction and step size resolution of 1 nm. The samples were mounted on an XYZ manual stage. The in-plane optical resolution was approximately 500 nm with the 730 nm excitation. The excitation laser was reflected by a dichroic mirror (DM) single edge at 785 nm (Di02-R785-25x36 from Semrock (Rochester, New York, NY, USA), transmission >93% between 804.3 and 1200 nm) and the collected PL was transmitted back to the detection arm and filtered using a Thorlabs Inc. (Newton, NJ, USA) 850 nm long-pass (LP, FEL0850). The fluorescence was collected using an achromatic aspherical converging lens of 100 mm focal length and fiber launcher connected to a multimode 1 m patch fiber coated from 700 to 1500 nm, with 62.5 μm core used as the confocal aperture. The photons are then sent to a single photon Si avalanche photodiode. A time correlator card (PicoQuant GmbH, Berlin Germany TimeHarp 260) was used to obtain time-resolved PL decay traces using the Fianium repetition rate of 20 MHz.

The second confocal is similar but with all optics optimized for IR emission, a CW 976 nm diode laser was used to excite the SiC samples and perform confocal mapping, a DM at 980 nm (Di02-R980-25x36), and a long bandpass (LP) filter at 980 nm (BLP01-980R-25) were used. The Olympus dry objective (100×, 0.85 NA) LCPLN-IR was used and the PL was measured in the IR using 10% of the emission from the color centers using a Spectrometer Princeton with a PyLoN-IR camera liquid nitrogen cooled to −110 °C.

Room and low temperature micro-PL were performed using the Micro-Raman Renishaw inVia (Wotton-under-Edge, Gloucestershire, UK) system spectrometer, equipped with a laser at 532 nm. The laser is focused via a 100× objective. The emitted PL is collected with the same objective and spectrally resolved in a spectrometer in Czerny-Turner configuration. A Peltier-cooled silicon-based charge-coupled device (CCD) is used as line-detector for (200 nm to 1050 nm) wavelength range.

## 4. Results and Discussion

### 4.1. Laser Writing at 515 nm

#### 4.1.1. High Purity Semi-Insulating 4H-SiC: Confocal Maps

**Figure 2:**
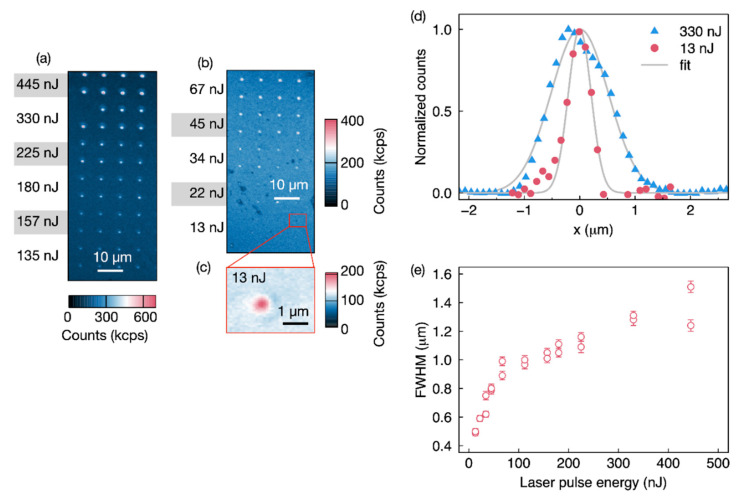
Confocal maps of high-purity semi-insulating (HPSI) 4H-silicon carbide (SiC) containing arrays of color centers laser written with a 515 nm fs-laser at single pulse energies ranging from (**a**) 135-445 nJ to (**b**) 13–67 nJ. (**c**) It shows a zoom in of the 13 nJ spot. (**d**) Point spread function for laser written spots created with 13 and 330 nJ per pulse. Fit is with a Gaussian profile. The confocal excitation laser is at 730 nm and 5 mW, while the collection is above 850 nm. (**e**) Lateral (X-direction) full with half maximum (FHWM) of the fabricated areas versus laser pulse energy.

**Figure 3:**
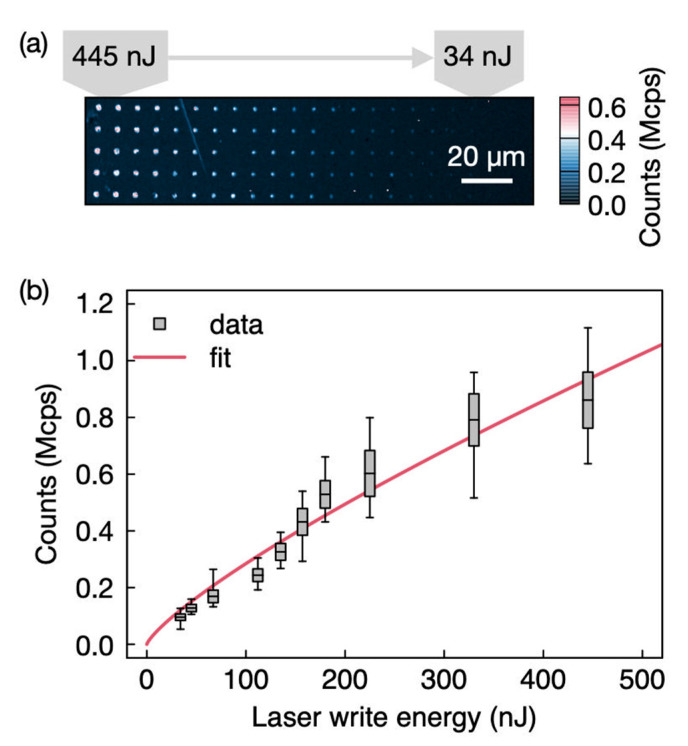
(**a**) Confocal map of color centers in HPSI 4H-SiC where the laser writer is focused 4 μm beneath the surface. A 515 nm fs-laser with single pulse energies ranging from 13 to 445 nJ was used. The confocal microscope laser was a 730 nm laser set to 1.2 mW and collection is above 850 nm. (**b**) The mean count rate of the laser written spots versus the laser writing energy. Background correction is not performed. The vertical extent of the boxes marks the standard deviation. The error bars denote the maximum and minimum values. The solid line is a power law ($PL\propto E_{p}^{k})$ fit excluding the two highest data points, with k=0.80 ± 0.07.

**Figure 4:**
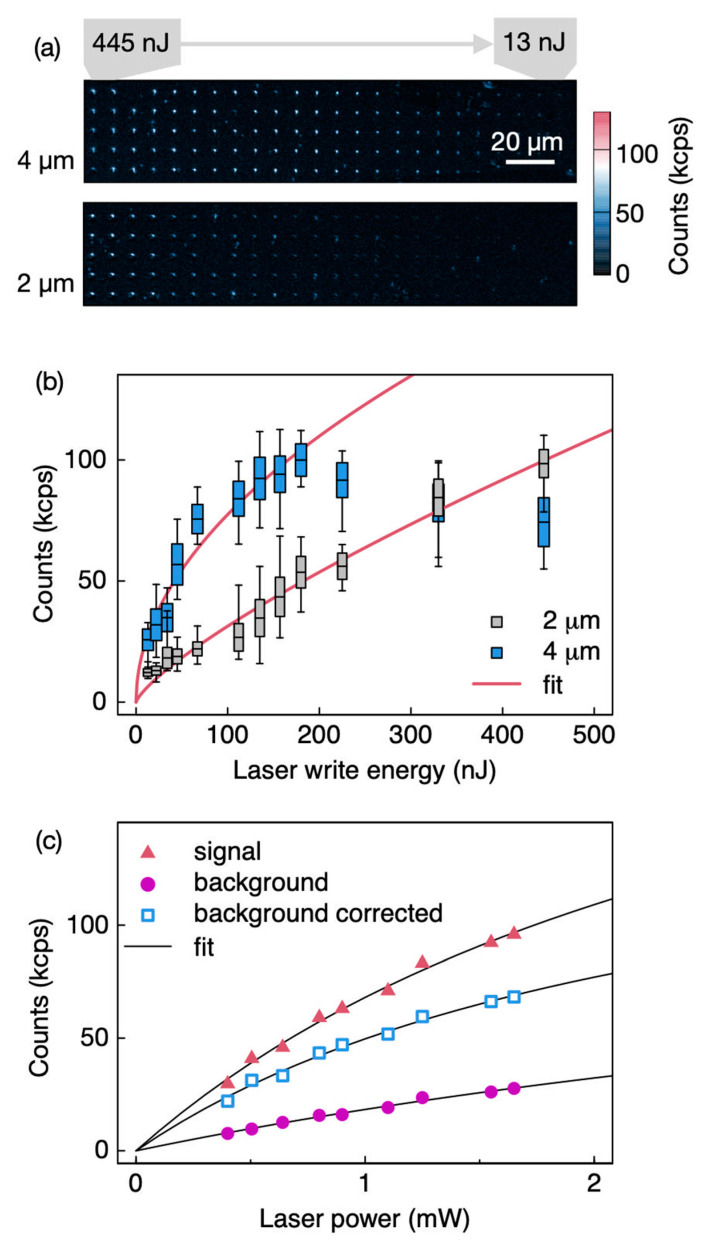
(**a**) Confocal maps of 515 nm fs laser written n-type 4H-SiC prepared at depths of 4 μm (top panel) and 2 μm (bottom panel). The confocal laser was 976 nm and 2 mW, and a collection was above 980 nm. (**b**) The PL intensity as a function of the laser write energy. Fit with power law $PL\propto E_{p}^{k},\;k=0.77\;\pm \;0.05$. (**c**) PL intensity for a laser written spot at 4 μm versus the confocal laser optical power at 976 nm showing a saturation with $\phi_{sat\;}=172$ kcounts/s and $P_{sat}\;$ = 2.5 ± 0.4 mW.

The fluorescence confocal maps of a HPSI 4H-SiC laser written using 515 nm with single shot pulse with energy steps from 445 to 13 nJ at two focal depths of 2 (Figure 2) and 4 μm (Figure 3) are shown. Imaging is done using a 730 nm excitation at 5 and 1.2 mW, respectively, with DM at 785 nm, LP at 850 nm, and confocal objective with a NA = 0.85, optimized for photons collected above 900 nm.

The samples were not annealed after laser irradiation.

In the confocal maps in Figure 2, each horizontal line of the array was created with the same energy/pulse as indicated. Laser pulse energies below 13 nJ did not provide any PL suggesting that the damage was not enough to produce vacancies or localization was not achieved.

For this sample, the estimated threshold intensity to create vacancies is $I^{th}=$ 70.4 TW/cm^2^ and 68 nJ/pulse, as such a lower energy should provide a much lower number of vacancies. The confocal map of an individual spot written at 330 nJ/pulse has a full with half maximum (FHWM) in the lateral (XY) direction of 1.22 ± 0.03 and 1.25 ± 0.03 μm, while at 13 nJ, the spot had a FWHM of 498 ± 2 nm (Figure 2d). The fabrication spots at lower energy are comparable with the confocal lateral diffraction limit of 0.61 λ/(2 NA) = 494.8 nm. The FWHM of the written spots versus energy is provided in Figure 2e.

Figure 3a presents a representative confocal image of a fs-laser written array of defects in HPSI 4H SiC. The spots are arranged in columns formed with the same laser energy. Energies of 445 nJ (left) to 34 nJ (right) were investigated. Figure 3b presents the average count rate of the spots obtained from 40 fabricated spots in each column (confocal maps up to 200 × 200 µm^2^ were collected) versus the laser pulse energy, $E_{p},$ showing good agreement with a power law characteristic of the MPI process.

We observe a lack of a strong nonlinearity of the trend in Figure 3b, for instance, it was previously reported [14] that the number of color centers created is related to a multiphoton absorption statistical probability with *k* = 16. We attribute this to other optical effects emerging from the confocal spectrally integrated measurements, where the emission is collected above 850 nm. At room temperature, the emission from shorter wavelength defects, e.g., the broad center at 770 nm can leak into the filtered bandwidth. As such low temperature studies centered on the zero-photon line (ZPL) of the target vacancy defect, *V*1′ is required. Otherwise, measurements with color center densities of the order of a few should be performed to improve the accuracy. Further, at high energy pulses some scattering due to the ablated region is also present, which could be responsible for the departure from the expected nonlinear trend. Finally, the sample has a relevant background from the as grown *V*_Si_ See Section 4.1.3 for deatils), which is actually unusual from this type of material grade. By subtracting the background, we have obtained *k* = 1.39 ± 0.04 and 1.49 ± 0.07 for the 2 and 4 µm depths, respectively.

It has also to be noted that the MPI or tunneling breakdown models combined with impact ionization and heat dissipation induce the generation of energetic seed electrons, while the vacancy formation mechanism is not yet fully understood. For example, exciton self-trapping could provide the energy necessary to initiate localized vacancy formation as otherwise electronic excitation would remain completely delocalized.

Finally, due to the tight focusing with NA = 0.9, self-focusing in air and subsurface region of samples was negligible. Filamentation of intense fs-pulses in air was also absent at the used irradiance.

From the saturation count rate at 1.2 mW, considering that a single color center was measured in the 4000 counts/s [14], here, for the fabrication at 4 µm and 13 nJ, we have a count rate of ~22,500 counts/s (after background subtraction), that is about 5–6 *V*_Si_ color centers, while at 445 nJ, we obtain ~788,000 counts/s and we estimate 190–200 emitters.

#### 4.1.2. *n*-Type 4H-SiC: Confocal Maps

The confocal map of the *n*-type SiC, studied with 976 nm excitation, DM at 980 nm, LP filter at 980 nm, and objective NA = 0.85, is shown in Figure 4a. No annealing is performed in this sample. PL was measured down to the 13 nJ/pulse with localization within the confocal diffraction limit of ~664 nm. In addition, for this fabrication, the average spot count rate versus pulse energy follows a power law for laser pulse energies less than 113 nJ with a lower dependence from the laser fabrication pulse energy, while at high fabrication energies there is a saturation and reduction in PL as the laser energy increases, suggesting a different damage mechanism and a different color center fabricated than the *V*_Si_ (Figure 4b). As the samples were not annealed, this emission could be a damage-related defect. In Figure 4c, the count rate, Ф, of a single spot in (a) is shown versus the optical power of the 976 nm confocal laser, $P_{opt},$ well fitted by a saturation function as $\phi =\frac{\phi_{sat}P_{opt}}{\left(P_{opt}+P_{sat}\right)}$, indicative of color center emission, while the background follows a linear dependence.

#### 4.1.3. PL from High-Purity Semi-Insulating 4H-SiC

**Figure 5:**
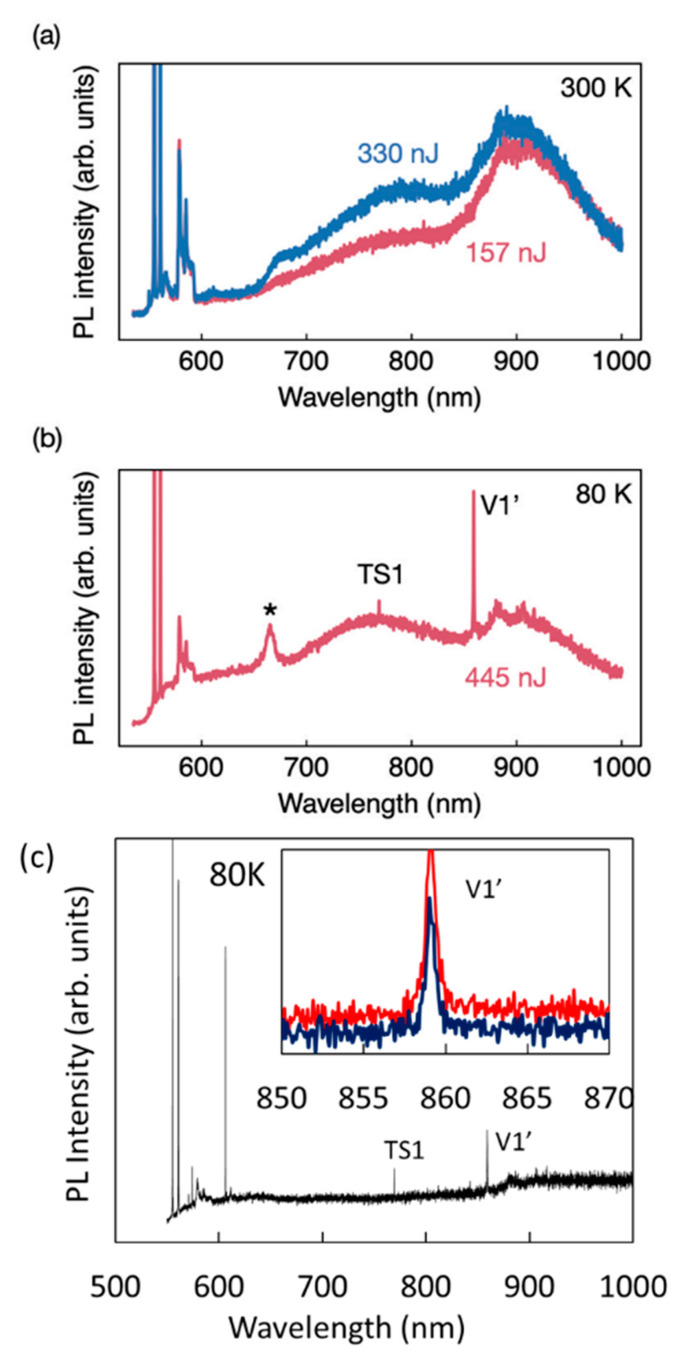
Spectroscopy of the (**a**) HPSI 4H-SiC laser written area corresponding to the highest energy dots of 445 and 330 nJ showing a broad emission centered at 920 nm and an emission at 770 nm. (**b**) Here, 80 K spectroscopy of a dot at 445 nJ. *V*1′ is at 859 nm corresponding to the hexagonal ZPL of the *V*_Si_ in the 4H-SiC. The emission at 769 nm shows a ZPL at low temperature, TS1, previously observed in proton irradiated 4H-SiC annealed at high temperature [45] however, the origin is unknown. A peak indicated with (*) is also unknown emission and it is due to laser irradiation. (**c**) Further, 80 K spectroscopy of the area not fabricated showing a background of the TS1 and *V*1′ line. The *V*1′ line was enhanced after laser fabrication as shown in the inset, where the emission in red is from the laser fabricated area. Excitation is at 532 nm, and collection is above 532 nm.

Using room temperature and 80 K spectroscopy, we determined the origin of the PL as shown in Figure 3. The broad emission of the fabricated areas at 920 nm is attributed to the *V*_Si_ [43,44] (Figure 5a). In addition, for fabrication at 2 μm, we observed also a room temperature broad emission at 770 nm, which is attributed to surface damage.

At lower temperature, the *V*_Si_^(−)^ two zero phonon lines (ZPLs) *V*1/*V*1′ at 862 and 859 nm, respectively, and *V2* at 917.5 nm in the 4H-SiC can be detected, with most evident the *V*1′ for c-axis grown material. These lines correspond to the two inequivalent sites, cubic (*V*2) and hexagonal (*V*1/*V*1′) sites, in 4H-SiC, with *V*1′ the second excited state of the hexagonal site. The intensity from a single defect at saturation is ~4–10 kcps [14,44] in bulk material using a-Si detector with 20–30% quantum efficiency. As the sample here fabricated had a large background (Figure 5c), *V*_Si_ single defects could not be assessed.

At 80 K (Figure 5b), we observed from the fabricated area a clear emission from the *V*1′ at 859 nm corresponding to the optical transition between the ground state and the second excited state of the *V*_Si_ center, as also observed in ref. [14], while *V*1 and *V*2 are very weak. Since, in our optical arrangement, we collect fluorescence parallel to the c-axis of 4H-SiC, we cannot observe much emission from the first excited state *V*1 and from *V*2, whose dipoles orientation are along to the c-axis. Nevertheless, as we have produced ensemble of emitters, the *V*2 line is also produced. There is a negligible broadening of the *V*1′ line from the fabricated area and the pristine area (Figure 5c inset) indicating a low residual damage even at 445 nJ laser fabrication. Additional peaks of unknown origin in the visible are observed at 80 K, which are associated to laser fabrication.

### 4.2. Laser Writing at 1030 nm

#### 4.2.1. GaN

We have compared the laser fabrication using 515 nm with 1030 nm in GaN and 4H-SiC.

**Figure 6:**
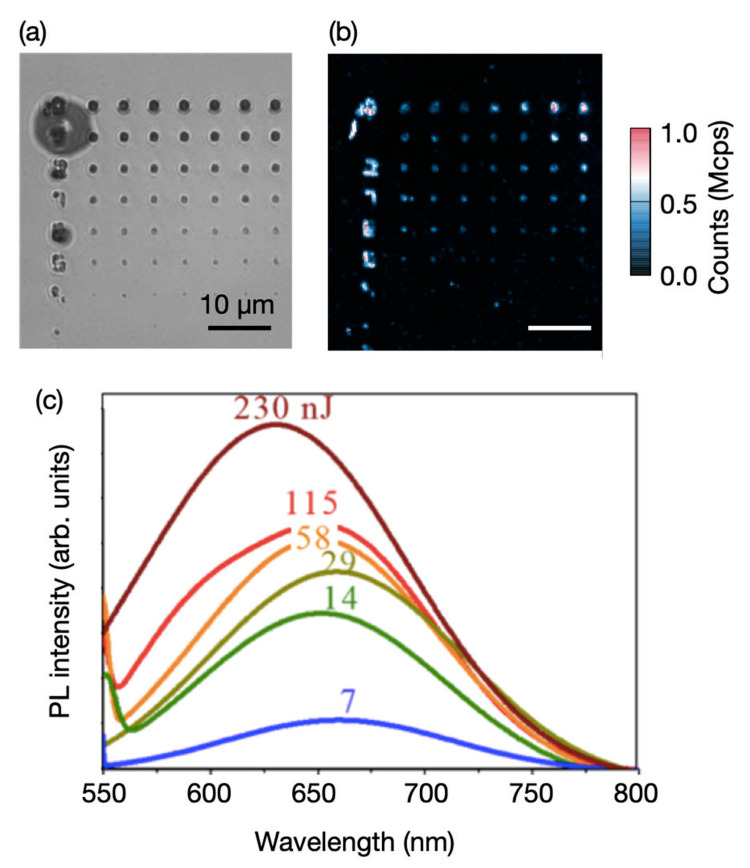
Gallium nitride (GaN) sample laser written using 1030 nm fs laser. (**a**) Bright field image using an oil objective 1.4 NA. (**b**) Confocal map with excitation at 532 nm, dichroic mirror (DM) 532 nm and short pass filter at 842 nm. (**c**) Photoluminescence (PL) spectra of typical emission at the observed energy writing pulses, showing a blue shift of the peak wavelength for lower energy pulses.

We show in Figure 6a, the fabrication of GaN close to the surface using 1030 nm. In Figure 6, a confocal map of laser written color centers in a 15 μm thin film of Wurtzite GaN (w-GaN) grown on top of a sapphire substrate via MOCVD is shown. The PL is observed up to 7 nJ/pulse using 532 nm excitation and a short pass filter at 842 nm. However, the fabrication shows irregular PL spots not diffraction limited using 532 nm for confocal imaging. As well for GaN, the PL of the fabricated area had a very small dependence on the laser energy and an emission blue-shifted for higher energy laser pulses. The PL suggests similar centers studied in ref. [18], that could be associated to RL centers related to *V*_Ga_ complex [32]. No annealing was performed in this sample.

#### 4.2.2. Semi-Insulating Unintentionally Doped 4H-SiC

**Figure 7:**
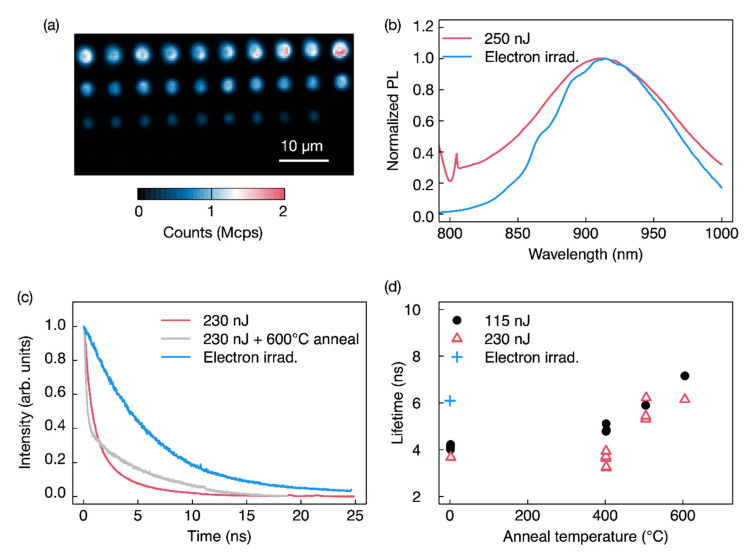
Semi-insulating unintentionally doped 4H-SiC laser written close to the surface using 1030 nm fs laser after 400 °C annealing. (**a**) Confocal excitation 730 nm, DM 785 nm, and long pass filter at 850 nm; the bright spots are associated to *V*_Si_. (**b**) Room temperature PL of the 230 nJ laser written spot compared to an electron-irradiated sample. A spectral broadening and shift are observed for the laser written emission. Excitation is at 785 nm, and collection is above 800 nm. (**c**) Exemplary lifetime decay for the dots fabricated at 230 nJ with no annealing and 600 °C annealing, compared to an electron-irradiated PL lifetime in a similar substrate. (**d**) Lifetime of the emission at the laser fabricated area versus annealing temperature.

We show as comparison, the fabrication in semi-insulating and unintentionally doped 4H-SiC using the approach as described in ref. [15], with the laser fabrication wavelength of 1030 nm and *NA* = 0.9. The confocal map revealed fluorescence only up to 58 nJ/pulse fabrication (Figure 7a) and the localization was limited by a larger writing volume of 1.9 μm^3^ giving a localization of the emission at 58 nJ of ~1 µm using 730 nm excitation. This is a reduced localization compared to the fabrication here shown in Figure 2 at 515 nm. As such 515 nm laser fabrication contributes to a better localization and reduced damage approaching the MPI regime.

In Figure 7a, the confocal map bright spots are from emission of *V*_Si_ as shown from room temperature PL. In Figure 7b, a PL comparison at room temperature with an electron-irradiated semi-insulating 4H-SiC (2 MeV fluence and density of 10^18^ e/cm^2^) is also provided, showing a broadening of the laser written *V*_Si_ emission.

In this sample, we also performed annealing steps in forming gas (Argon) for 30 min, showing a dependence of the fabricated color centers optical transition lifetime attributed to the *V*_Si_ with annealing temperature. The PL decay lifetime is compared with the optical transition lifetime of 6.1 ± 0.1 ns of the same emitters fabricated by electron irradiation. While the electron fabricated emission fluorescence decay can be fit by a single exponential, the laser written emission decay is modelled by two exponentials as shown in Figure 7c,d with a fast (<1 ns) and a slower (>3.2 ± 0.1 ns) decay. The lifetime associated to the slower decay increases to 6.2 ± 0.1 ns with 600 °C annealing. The different lifetime behavior of the *V*_Si_ with the two fabrication methods may be due to an higher formation of the carbon vacancies [46], *V*_C_, by laser writing compared to the electron irradiation. While the *V*_C_ is not fluorescent, it can create nonradiative pathways for *V*_Si_ thus quenching the fluorescence. Quenching mechanism of *V*_Si_ creation were also observed in proton-writing of *V*_Si_ [47,48] and laser writing [14]. Annealing at moderate temperature may reduce the concentration of the V_C_ and improve the radiative lifetime time of the emitters, however, high-temperature annealing also convert the *V*_Si_ in *C*_Si_*V*_C_ [49] and divacancy [50] and possibly other defects are also formed [45].

## 5. Conclusions

In summary, we have demonstrated that 515 nm femtosecond laser writing can provide a better control of the localization and less damage in the creation of the *V*_Si_ in SiC, with a localization within the imaging diffraction limit and showing a power law dependence of the number of color centers with the laser pulse fabrication energy. The fabrication at 515 nm does not introduce spectral broadening compared to the as grown *V*_Si_ in the material as observed from the 80 K PL. By selecting a lower background substrate, large array of these color centers can be fabricated and studied for quantum sensing and single photon sources [51]. It is shown how the fabrication of the color centers depends also on the original doping of the material as high n-doped SiC provides emission at longer wavelength.

We compare our results with the state-of-the-art fabrication of color centers using femtosecond lasers achieving high localization in Table 2. Table 2 also reviews a variety of conditions adopted to produce color centers in diamond, SiC, GaN, and cBN. We show that direct laser writing can be employed in ultrapure, semi-insulating, and n-doped 4H-SiC and in unintentionally doped GaN. Controlled arrays of luminescent emission in the red and near infrared regions localized within the diffraction limit are shown. The observed emissions are attributed to the *V*_Si_ in 4H-SiC using low temperature PL, while the emission at longer wavelength is not currently identified. High localization with higher energy/pulse without the use of SLM can be achieved by adjusting the repetition rate, the writing laser wavelength, and the NA of the focusing optics.

In comparison to the previous use of 1030 nm for fabrication, we have achieved a better localization and control of the number of color centers without the use of a SLM. Future studies may involve annealing steps experiments of the laser fabricated array also using multiple laser pulses [28] to determine the conversion of the color centers produced in other relevant color centers in SiC such as the carbon antisite vacancy pairs [49,53], divacancy [50,54], and nitrogen vacancy centers [55,56]. The fabrication has also created nanometric size craters on the surface and nanometric debris showing fluorescence when excited at 730 and 976 nm. Studies on the effect of nanoablation regime in relation to color centers formation in SiC could be of interest for the future. The immediate application of these methods is in a facile and more deterministic on demand generation of key color centers in SiC for single photon sources and distributed quantum networks [57].

## Figures and Tables

**Table 1:** Samples studied in this work.

λ	$E_{p}$ nJ Per Pulse	Material	Color Centers Identified and Depth (μm)	Annealing
nm				
515	13 ÷ 445	4H-SiC	*V*_Si_ 665 nm unknown (2, 4)	Before fabrication at 1000 °C
		HPSI		
		N < 10^14^ cm^−3^		
515	13 ÷ 445	4H-SiC	Unknown (2, 4)	Not performed
		n-type		
		N 10^19^ cm^−3^		
1030	4 ÷ 230	GaN	RL center (0)	Not performed
		Thin film		
		unintentionally		
		n-doped		
1030	4 ÷ 230	4H-SiC	*V*_Si_ (0, 5, 10)	400, 500, and 600 °C
		Unintentionally doped		
		N 3–6 × 10^15^ cm^−3^		

**Table 2:** Laser fabrication conditions for various experiments with laser wavelength λ, waist $w_{o}$, objective numerical aperture NA, theoretical pulse energy and laser irradiance determining MPI ($E_{p}^{th}$ and $I^{th}$), laser pulse width and repetition rate, τ and $R_{p}$, respectively, energy per pulse used in the experiments, and $E_{p}$, material type and color centers generated with depth localization.

λ	$I^{th}$	$E_{p}^{th}$	$w_{o}$	NA	τ	$R_{p}$	$E_{p}$	Material	Color Centers Identified and Depth	Reference
nm	TW/cm^2^	nJ	nm		fs	kHz	nJ Per Pulse		(μm)	
790	13.4	12.9	350	1.4	250	1	6.7 ÷ 89.9	4H-SiC ^1^	*V*_Si _^5^	[14]
								Intrinsic	−40	
1030	17.7	68	729	0.9	230	200	29 ÷ 230	4H-SiC ^2^	*V*_Si_, *V*_c_*V*_Si_	[15,52]
								intrinsic	(0, 5, 10)	
515	70.9	68	364	0.9	230	200	13 ÷ 445	4H-SiC	*V*_Si_	This work
								HPSI	(2, 4)	
515	70.9	68	364	0.9	230	200	13 ÷ 445	4H-SiC	Unknown	This work
								n-type ^3^	(2, 4)	
515	57.5	22.8	234	1.4	230	200	9 ÷ 90	cBN	NV	[16]
									(10, 20)	
1030	16.6	63.8	729	0.9	230	200	4 ÷ 230	GaN	RL center	This work
								Thin film	0	
800	27.6	74.4	784	0.6	140	80,000	1.6 ÷ 5.74	GaN	RL centers	[18]
								Thin film	0	
790	26.6	25.6	350	1.4	250	1	19.6 ÷ 61.8	Quantum	NV ^5^	[10]
								Diamond ^4^	−40	
515	62.6	31.1	262	1.25	230	500	10 ÷ 30	Quantum	NV ^5^	[12]
								Diamond	−25	
800	25.9	67.7	1020	0.5	80	250	4,000 ^3^	Type Ib	NV ^5^	[40]
								Diamond	(around crater)	
800	25.9	13	566	0.9	50	1	10,000 ^3^	Quantum	SiV ^5^	[11]
								Diamond	(around crater)	

^1^ Nitrogen doping of 1 × 10^15^ cm^−3^, ^2^ Nitrogen doping of 3–6 × 10^15^ cm^−3^, ^3^ Nitrogen doping of 10^19^ cm^−3^, ^4^ Electronic grade diamond, ^5^ Studies showing single color center fabrication.

## Data Availability

The data presented in this study are available on request from the corresponding author.
